# Bile Acids and Their Derivatives as Potential Modifiers of Drug Release and Pharmacokinetic Profiles

**DOI:** 10.3389/fphar.2018.01283

**Published:** 2018-11-08

**Authors:** Nebojša Pavlović, Svetlana Goločorbin-Kon, Maja Ðanić, Bojan Stanimirov, Hani Al-Salami, Karmen Stankov, Momir Mikov

**Affiliations:** ^1^Department of Pharmacy, Faculty of Medicine, University of Novi Sad, Novi Sad, Serbia; ^2^Department of Pharmacology, Toxicology and Clinical Pharmacology, Faculty of Medicine, University of Novi Sad, Novi Sad, Serbia; ^3^Department of Biochemistry, Faculty of Medicine, University of Novi Sad, Novi Sad, Serbia; ^4^Biotechnology and Drug Development Research Laboratory, School of Pharmacy and Biomedical Sciences, Curtin Health Innovation Research Institute, Curtin University, Perth, WA, Australia

**Keywords:** bile acids, pharmaceutical formulation, drug delivery, bioavailability, physical complexation, drug cellular transport

## Abstract

Bile acids have received considerable interest in the drug delivery research due to their peculiar physicochemical properties and biocompatibility. The main advantage of bile acids as drug absorption enhancers is their ability to act as both drug solubilizing and permeation-modifying agents. Therefore, bile acids may improve bioavailability of drugs whose absorption-limiting factors include either poor aqueous solubility or low membrane permeability. Besides, bile acids may withstand the gastrointestinal impediments and aid in the transporter-mediated absorption of physically complexed or chemically conjugated drug molecules. These biomolecules may increase the drug bioavailability also at submicellar levels by increasing the solubility and dissolution rate of non-polar drugs or through the partition into the membrane and increase of membrane fluidity and permeability. Most bile acid-induced effects are mediated by the nuclear receptors that activate transcriptional networks, which then affect the expression of a number of target genes, including those for membrane transport proteins, affecting the bioavailability of a number of drugs. Besides micellar solubilization, there are many other types of interactions between bile acids and drug molecules, which can influence the drug transport across the biological membranes. Most common drug-bile salt interaction is ion-pairing and the formed complexes may have either higher or lower polarity compared to the drug molecule itself. Furthermore, the hydroxyl and carboxyl groups of bile acids can be utilized for the covalent conjugation of drugs, which changes their physicochemical and pharmacokinetic properties. Bile acids can be utilized in the formulation of conventional dosage forms, but also of novel micellar, vesicular and polymer-based therapeutic systems. The availability of bile acids, along with their simple derivatization procedures, turn them into attractive building blocks for the design of novel pharmaceutical formulations and systems for the delivery of drugs, biomolecules and vaccines. Although toxic properties of hydrophobic bile acids have been described, their side effects are mostly produced when present in supraphysiological concentrations. Besides, minor structural modifications of natural bile acids may lead to the creation of bile acid derivatives with the reduced toxicity and preserved absorption-enhancing activity.

## Introduction

Oral delivery is regarded as the preferred route of drug intake considering the ease of administration and the patients’ acceptance. However, the gastrointestinal tract represents the substantial physical and a biochemical barrier to the systemic availability of orally ingested medicines due to harsh acidic environment in the stomach and enzymatic degradations, variable pH in the intestine, mucus secretion, etc. (Stojančević et al., 2014). Besides, the oral bioavailability of drugs depends on many factors, primarily on aqueous solubility and dissolution rate, permeability across biological membranes, pre-systemic metabolism and susceptibility to efflux mechanisms (Gomez-Orellana, 2005).

Approximately 60–70% of all drug molecules are insufficiently soluble in aqueous media and/or have very low permeability to be adequately absorbed from the gastrointestinal tract following oral administration (Gupta et al., 2013). Poor aqueous solubility of lipophilic drugs impacts the dissolution rate and subsequently the drug absorption, since dissolution needs to be completed within the intestinal transit time limit to maximize drug absorption and only dissolved drug fraction may gain access to the surface of absorptive cells (Jain et al., 2015). On the other hand, too hydrophilic drugs are often poorly absorbed because of their inability to cross the lipid-rich cell membranes. For a drug to be readily absorbed by passive diffusion, it must be largely hydrophobic, yet have sufficient solubility in aqueous solutions within the physiological range of pH (Liu et al., 2011). However, molecules can be transported actively across the membrane as well, *via* specialized transport proteins, and transporter-mediated transfer of a compound from one compartment to another may influence its pharmacokinetics. Highly hydrophilic compounds may have good bioavailability when absorbed by uptake transporters, while some hydrophobic drugs may be poorly absorbed if they are substrates for efflux membrane transporters (Nielsen et al., 2010).

Various strategies have been investigated to enhance the bioavailability of poorly absorbed drugs, including chemical modifications, the use of novel excipients, drug carriers, enzyme inhibitors, absorption enhancers, etc. The formulation design is usually the approach of choice, particularly for drugs that are already in development stages or on the market, since it doesn’t require chemical modifications or synthesis of new chemical entities (Gomez-Orellana, 2005). The use of absorption enhancers is the simplest approach to enhance the drug permeation across the intestinal epithelium (Williams and Barry, 2004).

The excipients used as carriers for drug delivery should meet the requirements of biocompatibility and biodegradability, as well as enabling adequate drug loading and controlled drug release. Bile acids are biomolecules that have received considerable interest in drug delivery research due to their biological compatibility and favorable toxicity profiles (Nurunnabi et al., 2016). The advantage of bile acids as drug absorption enhancers is their ability to act as both drug solubilizing and permeation-modifying agents. Therefore, bile acids may improve bioavailability of drugs whose absorption-limiting factors include either poor aqueous solubility or low membrane permeability (Pavlović et al., 2017). Besides, the most of absorption enhancers use the paracellular pathways or the rupture of tight junctions to deliver the drug molecules, which is often insufficient in comparison to transporter-mediated active transport. As bile acids are efficiently absorbed *via* the apical sodium-dependent bile acid transporter (ASBT) in the ileum, this protein may also serve as a target for improving oral bioavailability of poorly absorbed drugs that are chemically conjugated or physically complexed with bile acids (Nurunnabi et al., 2016).

The aim of this study is to provide an overview of the current knowledge related to the role of bile acids, their salts and novel derivatives in modification of drug release and pharmacokinetics, strategies for improving oral bioavailability of drugs through design of bile acid-based formulations and to evaluate toxicological aspects of the application of bile acids.

## Physicochemical Properties of Bile Acids

Bile acids are physiological surfactants that are synthesized from cholesterol in the liver. In humans, two primary bile acids, cholic acid (CA) and chenodeoxycholic acid (CDCA), are synthesized in the liver, and they are conjugated to either taurine or glycine at the C-24 carboxyl group before active secretion *via* the canalicular membrane of hepatocytes. Intestinal bacteria metabolize bile salts during their enterohepatic circulation and the major biotransformation reactions include hydrolysis of conjugated bile acids to free bile acids by bile salt hydrolase, and 7α-dehydroxylation of CA and CDCA yielding deoxycholic acid (DCA) and lithocholic acid (LCA), respectively. Ursodeoxycholic acid (UDCA) is formed by the 7β-epimerization of CDCA in the large intestine through reactions mediated by enteric bacteria enzymes (Ridlon et al., 2016).

Bile acids have specific chemical structure, characterized by a large, rigid, and planar hydrophobic steroid nucleus with hydroxyl groups varying in number, position and orientation, along with a flexible acidic side chain. Bile acid molecules are approximately 20 Å long, with an average radius of about 3.5 Å (Monte et al., 2009). Due to their weak acid properties, bile acids exist in ionized form as bile salts at physiological conditions. The pKa values of unconjugated bile acids are approximately 5, whereas the conjugation with amino acids further lowers pKa to values around 4 in glycine conjugates and less than 2 in the taurine conjugates. Therefore, the bile acid amidation leads to the improvement of their physicochemical properties, including increased hydrophilicity and aqueous solubility, decreased cytotoxicity and enhanced resistance to precipitation due to low pH or the presence of divalent cations such as Ca^2+^ during digestion (Hofmann and Hagey, 2008). Conjugated bile acids thus represent slightly stronger acids with lower pKa values, and taurine-conjugated bile acids are soluble even at gastric pH values and are thus suitable for designing oral drug delivery systems (Nurunnabi et al., 2016).

**FIGURE 1:**
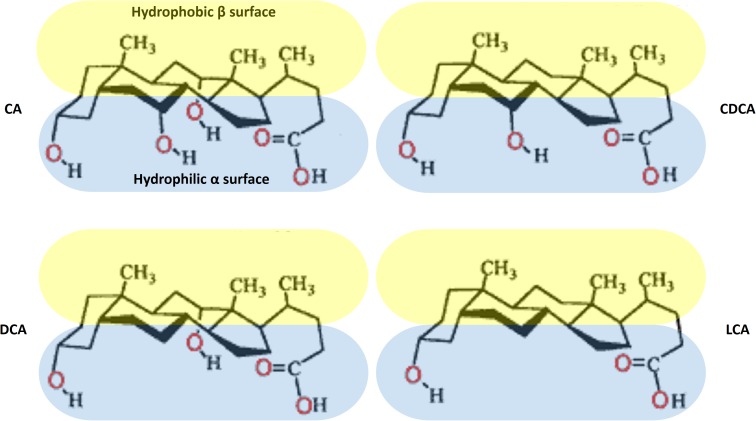
Chemical structures of the main natural bile acids in humans.

The natural bile acids are the derivatives of 5β-cholanic acid with *cis* A–B ring junction, which results in a slight curvature of the steroid skeleton. Therefore, bile acid molecules are characterized by two clear-cut hemispheres: a convex, hydrophobic β side containing angular methyl groups at positions C-18 and C-19, and a concave, hydrophilic α side containing 1–3 polar hydroxyl groups (Bukiya et al., 2008). The orientation of the polar groups to one hemisphere confers a facial amphiphilicity of bile acids, in contrast to conventional surfactants that contain clearly separated polar head group and a long non-polar hydrocarbon chain. The exception is UDCA that contains a hydroxyl groups on both α- and β-side and therefore has increased hydrophilicity (Martínez-Augustin and de Medina, 2008). The chemical structures of the main unconjugated natural bile acids in humans are presented at Figure 1.

Bile acids as facial amphiphiles contain specific surface active and interfacial properties. They have been suggested to orient at the oil-water interface with the steroid backbone parallel to the interface, allowing the hydroxyl groups to interact with water molecules. However, due to less efficient packing at the interface compared to conventional surfactants, bile acids attain higher surface tension values in aqueous solutions (Maldonado-Valderrama et al., 2008). It was demonstrated that increased hydrophobicity of bile acids, as exemplified by obtaining DCA from CA through the removal of a hydroxyl group, led to a higher affinity for the oil-water interface and more efficient interfacial protein displacement (Euston et al., 2011).

Due to their amphiphilic properties, bile acids can self-associate in water, thus forming supramolecular aggregates or micelles, when their concentration is above a certain concentration termed the critical micellar concentration (CMC). Naturally occurring bile acids have CMCs in the range of 2–20 mM in water, which is in accordance with their aqueous solubility at body temperature. As a consequence of the rigid molecular framework and planar polarity, bile acids tend to form smaller micelles with low aggregation numbers and display higher CMC values compared to those of conventional surfactants (Maldonado-Valderrama et al., 2011). CMCs of several natural bile acids and semi-synthetic derivatives are presented in the Table 1.

**Table 1:** Critical micellar concentrations (CMC) of natural bile acids and semisynthetic keto-derivatives.

Bile acid	Abbreviation	Position and orientation of OH groups	CMC [mM]
**Natural bile acids [18, 20]**			
Cholic acid	CA	*3*α *7*α *12*α	11–13
Chenodeoxycholic acid	CDCA	*3*α *7*α	4–9
Deoxycholic acid	DCA	*3*α *12*α	3–10
Ursodeoxycholic acid	UDCA	*3*α *7*β	7–19
Taurocholic acid	TCA	*3*α *7*α *12*α	6–10
Glycocholic acid	GCA	*3*α *7*α *12*α	10–12
Tauro-chenodeoxycholic acid	TCDCA	*3*α *7*α	3
Glyco-chenodeoxycholic acid	GCDCA	*3*α *7*α	2–6
Tauro-deoxycholic acid	TDCA	*3*α *12*α	2
Glyco-deoxycholic acid	GDCA	*3*α *12*α	2–6
Tauro-ursodeoxycholic acid	TUDCA	*3*α *7*β	2
Glyco-ursodeoxycholic acid	GUDCA	*3*α *7*β	4
**Semisynthetic keto derivatives [29]**			
12-monoketocholic acid	12-MKC	*3*α *7*α	67
7-monoketocholic acid	7-MKC	*3*α *12*α	68
7,12-diketocholic acid	7,12-DKC	*3*α	95
3,7,12-triketocholic acid	3,7,12-TKC	/	135
12-monoketodeoxycholic acid	12-MKDC	*3*α	20


Hydrophobicity is the most important determinant of self-assembly behavior of bile acids and their toxicity as well. The bile acid hydrophobicity increases in the following order: UDCA < CA < CDCA < DCA < LCA, with UDCA being the most hydrophilic and LCA the most hydrophobic natural bile acid. Hydrophilic–lipophilic balance of bile acids depends on the number, position and orientation of the hydroxyl groups, as well as conjugation with amino acids (taurine-conjugated < glycine-conjugated < free species) (Roda et al., 1990). The CMCs correlate inversely with hydrophobicity of bile acids, as measured by reverse phase HPLC retention factors, which demonstrates that, as expected, the driving force for micelle formation is to minimize the hydrophobic surface. Nevertheless, the conjugation of bile acids with glycine or taurine results in lower CMC values, which indicates that the complex interplay between the hydrophobic effect and specific hydrogen bonding interactions may contribute to the micellization and unexpected CMC values (Lucangioli et al., 2001; Madenci and Egelhaaf, 2010).

The stability of the bile acid micelles depends on both structural properties of bile acids and solution conditions, such as the temperature, pH and ionic strength. Generally, CMC values increase with the following structural modifications: the addition of hydroxyl groups, changing the orientation of a hydroxyl group from α- to β-side of the steroid backbone, replacing the hydroxyl group with keto (oxo) group, and shortening the side chain of conjugated bile acids (Holm et al., 2013). The reaction of 7β-epimerization of CDCA mediated by intestinal bacteria leads to the formation of UDCA, which has higher CMC value and reduced solubilization capacity for lipids in comparison to CDCA due to the presence of polar groups on both hemispheres of the bile acid molecule (Ridlon et al., 2006). CMC of bile acids decreases with increasing ionic strength, since electrolyte addition reduces repulsive electrostatic interactions between charged groups. Besides, reduction of pH to values close to the pKa of the bile acid lowers CMC as a consequence of partial protonation of bile acid anions, which become solubilized in bile salt micelles (Faustino et al., 2016).

As previously stated, two key forces, hydrophobic and hydrogen bonding interactions, determine the size and shape of bile acid aggregates and aid in the stabilization of micelles. Bile acids are able to self-assemble over a broad concentration range, which differs them from conventional surfactants. Namely, bile acids have been reported to have two CMC values and to form spherical micelles at the first CMC, whereas the structural transition from spherical to long rod-like micelles occurs at the second CMC value (Matsuoka et al., 2006).

The complex aggregation behavior of bile acids has been extensively studied and the two-step model for the process of the bile acids micellization has been widely accepted (Calabresi et al., 2007). The first step involves the formation of primary aggregates, which is driven by the hydrophobic interactions between hydrophobic surfaces of the monomers creating a hydrophobic cavity, with the polar groups pointing outwards. At higher bile acid concentrations, intermolecular hydrogen bonding between the hydrophilic groups of primary units create a central hydrophilic core, which is a complementary mechanism to the formation of secondary micelles. The ability of bile acid aggregates to encapsulate both small non-polar molecules inside the hydrophobic nanocavities of primary micelles and polar molecules inside the hydrophilic core of secondary micelles make them suitable for the design of advanced drug delivery systems (Li et al., 2009). Molecular dynamics simulations supported this two-step aggregation model based on formation of small, spherical or slightly aspherical oblate-shaped primary micelles consisting of 2–10 monomers held together by hydrophobic interactions, which associate further at higher concentrations to form complex objects of various shapes (secondary micelles) *via* hydrogen bonding (Pártay et al., 2007).

Structural modifications of natural bile acids have continuously being made in an attempt to reduce the cytotoxicity without reducing their ability to promote membrane permeability. It has been demonstrated that the replacement of hydroxyl with keto groups led to the significantly higher CMC values of bile acids and diminished membrane toxicity with preserved absorption-enhancing activity (Atanacković et al., 2009; Chen et al., 2012).

## Absorption-Enhancement Mechanisms Mediated by Bile Acids

Simple bile acid micelles are normally not formed *in vivo* in humans, but they aid in the solubilization of phospholipids and monoacylglycerols by forming mixed micelles. The CMC values of these mixtures are generally lower in comparison to bile acids alone, although depending on the ratio of phospholipids to bile salts (Marin et al., 2016). The inclusion of the lipophilic components lowers the CMC value and increases the size and solubilization capacity of the bile acid aggregates (Nanjwade et al., 2011). The solubilization potential of bile acids is of both physiological and pharmaceutical relevance, since bile acids in the intestinal lumen allow emulsification and absorption of dietary fats, liposoluble vitamins and lipophilic drug molecules as well, through the process of micellar solubilization. Conjugated bile acids have been shown to exert superior emulsification activity in comparison to unconjugated bile acids and to facilitate the absorption of lipids more efficiently.

Digestion of dietary lipids is a very complex multi-step process, initiating in the stomach, and ending in the small intestine. Physicochemical remodeling and lipase-catalyzed hydrolysis are the key events enabling efficient lipid absorption. When partially digested food enters the duodenum, bile and pancreatic juice are concomitantly secreted and mixed in the duodenum as a consequence of the hormonal stimulations. This significantly changes the medium properties, by providing high amounts of conjugated bile salts with strong detergent properties, digestive enzymes, especially pancreatic lipases and phospholipases, and high concentration of bicarbonates that raise the medium pH. The intestinal uptake of lipolytic products, mainly fatty acids, as well as free cholesterol and fat-soluble vitamins, is highly dependent on the efficiency of sequential actions by lipolytic enzymes on lipid substrates. Emulsification by bile salts in duodenum is the physicochemical process that enables the formation of oil-water interface and the surface access of lipolytic enzymes to their substrates. Lipolysis is mediated by the pancreatic colipase-lipase system which adsorbs onto the emulsion interface and catalyzes the cleavage of ester bonds on the triglyceride moiety, generating intermediate diglycerides and finally, fatty acids and monoglycerides. Besides, bile salts as components of intraluminal mixed micelles, subsequently solubilize lipid digests, enabling their access to the intestinal epithelium and improving their absorption (Lairon, 2009).

Lipophilic compounds, including drug molecules, with a low aqueous solubility are generally well solubilized in bile salt-phospholipid mixed micelles in the intestine, leading to their increased absorption and bioavailability. It has been shown that bile acids may act as absorption enhancers not only by increasing the solubility of hydrophobic drugs, but also by increasing the fluidity of biological membranes and promoting the chemical and enzymatic stability of drug molecules (Moghimipour et al., 2015). Therefore, bile acids may improve bioavailability of both non-polar drugs through the process of micellar solubilization, and highly hydrophilic drug molecules as well, through their interactions with biological membranes. The advantage of the use of bile acids as absorption enhancers is also their ability to withstand the gastrointestinal impediments and aid in the carrier-mediated absorption of drugs complexed or conjugated with bile acids (Nurunnabi et al., 2016).

**FIGURE 2:**
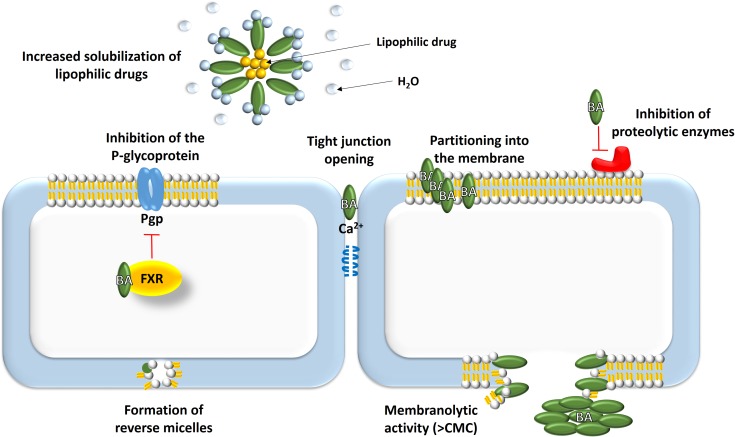
Absorption-enhancement mechanisms mediated by bile acids: (1) solubilization of lipophilic drugs; (2) effects on biological membranes – increasing fluidity, opening tight junctions, formation of reverse micelles, membranolytic effect, modulation of transport proteins; (3) improvement of chemical stability of drugs.

Permeation of a drug through a biological membrane by passive diffusion is influenced by the solubility and molecular weight of the drug molecule, the membrane structure and thickness of the mucus layer over the membrane. Penetration across a membrane is influenced by permeability, surface area and the concentration gradient (Mikov and Fawcett, 2006). Bile acids can increase the absorption of drugs that are otherwise unable to cross cell membranes and the main mechanisms for achieving these effects are presented at Figure 2.

The main strategy to enhance the absorption of lipophilic drugs is to increase their water solubility and dissolution rate, which can be achieved by micelle formation. Solubility studies showed that the aqueous solubility of rifaximin, practically water-insoluble and non-absorbable antibiotic drug, was increased 70- to 120-fold in the presence of the mixture containing equimolar physiological concentrations of six natural bile acids (2.5–20 mM) at pH 7.4. It was demonstrated that bile acids solubilized rifaximin, making it more bioavailable to inhibit the essential proteins required for bacterial growth and subsequently to exert its antimicrobial effect (Darkoh et al., 2010). Solubilization of non-polar drugs within bile salt mixed micelles may overcome the resistance at the aqueous boundary layer adjacent to the enterocytes, facilitating transcellular diffusion and improving the extent of absorption (Mikov et al., 2006). However, drug molecule needs to be liberated from bile salt micelles in order to pass across the membrane, since the intact micellar structure is impermeable. Micellar solubilization of drugs can therefore sometimes have also negative implications for bioavailability depending on the compound (Enright et al., 2018).

Bile acids are not equivalent with respect to their micellization capacity. The degree of steroidal ring hydroxylation was found to significantly impact the solubilization capacity of bile acids and therefore potentially the apparent solubility of drugs in the intestinal lumen. The solubilization capacity of the tauro-deoxycholate (TDCA) containing two hydroxyl groups was shown to be superior to the trihydroxy bile salt tauro-cholate (TCA) for all nine studied poor water-soluble drugs. It was concluded that more hydrophobic bile salts are more effective at micellization, while the solubilization capacity was inversely correlated to the surface tension lowering activity of bile salts (Enright et al., 2017). It should be noted that bile acids may increase the solubility and dissolution rate of non-polar drugs also at levels lower than the CMC, primarily through its ‘wetting’ effects, i.e., lowering the interfacial tension between a drug and the dissolution medium within which it is dispersed (Dulfer and Govers, 1995; Enright et al., 2018).

Bile salts may also act as crystallization inhibitors, which indicates their potential role in the formulation of supersaturating dosage forms or lipid-based drug delivery systems with a high proportion of co-solvent that may lose the solubilization power upon dilution in the intestine (Chen et al., 2015). The ability of 13 bile salts to maintain supersaturated aqueous solutions of lipophilic drugs was evaluated and it was shown that bile salts extended nucleation induction times. However, their inhibition effects varied depending on the structure of both the bile salt and the drug. For unconjugated bile salts, better crystallization inhibition properties were observed for bile salts with higher hydrophobicity, while the opposite trend was determined for glycine-conjugated bile salts (Li et al., 2016).

Besides their effects on drug dissolution, bile acids can interact with the phospholipid bilayer of biological membranes, causing the increase in membrane permeability and subsequent enhancement of absorption. At submicellar levels, bile acids can partition into the membrane and increase membrane fluidity and permeability, which is compound-dependent. They change the distribution of lipids and proteins in the membrane and thus can alter cell surface signaling through modulation of the stability of lateral domains (Zhou et al., 2013). On the other hand, bile acids at concentrations above their CMC may associate with phospholipids of cell membranes, causing the dissociation of integral membrane proteins and resulting in the breakdown of the cell membrane and subsequent cell death. This membranolytic effect of bile acids at high concentrations is directly related to the intensity of their enhancer effect (Garidel et al., 2007).

Bile acids can enhance transcellular transport of hydrophilic drugs by incorporating in cell membranes and producing reverse micelles, which results in the creation of hydrophilic pores, i.e., aqueous channels. Additionally, bile acids can increase the paracellular drug transport by binding calcium ions, thereby causing tight junctions between the cells to open. It is believed that filamentous actin has a significant role in the control of paracellular permeability. Moreover, bile acids can reduce the viscosity and elasticity of the mucus adhering to the epithelial surface and thus increase epithelial membrane permeability. It was also shown that bile salts may exert reversible and concentration-dependent inhibitory effects on mucosal membrane peptidases, and thus improve the stability and the following absorption of peptide drugs (Stojančević et al., 2013; Moghimipour et al., 2015).

Generally, the absorption-promoting activity is directly correlated with the hydrophobicity of bile acid molecule. However, several studies determined that hydrophobicity is not a crucial factor in transport efficacy. For example, trihydroxy TCA proved superior in promoting cholesterol uptake compared to dihydroxy tauro-chenodeoxycolate (TCDCA) despite a reduced solubilization capacity, which suggested that bile acids could affect cholesterol absorption through the transporter-mediated mechanisms (Nordskog et al., 2001).

Several bile salts, including UDCA, tauro-lithocholate (TLCA), tauro-chenodeoxycholate (TCDCA), glyco-chenodeoxycholate (GCDCA) and 12-monoketocholate (MKC), were shown to inhibit the active efflux of *P*-glycoprotein (Pgp) substrates, probably indirectly, by changing the lipid environment of the Pgp transporter or by interaction with Pgp itself. The absence of a hydroxyl group at position 12 is the only chemical determinant that is common for these bile acids, suggesting this is a decisive structural property for bile salts to inhibit Pgp (Yang et al., 2012). Numerous drugs, such as cytostatics vinblastine, paclitaxel, doxorubicin, epirubicin, etoposide, are substrates for Pgp efflux transporter and its inhibition may increase intracellular concentrations and activity of these drugs. It was demonstrated that glycocholic acid (GCA) increased the chemosensitivity of Caco-2 cells and rat intestine to epirubicin through the modulation of Pgp and multidrug resistance proteins, in addition to regulating apoptosis-related pathways (Lo et al., 2008).

Most effects induced by bile acids are mediated by the nuclear farnesoid X receptor (FXR) and the G protein-coupled receptor TGR5, but also by vitamin D receptor (VDR), pregnane X receptor (PXR), and constitutive androstane receptor (CAR). Nuclear receptors that respond to bile acids activate transcriptional networks and/or signaling cascades, which then affect the expression of a number of target genes, including those for transporter proteins (Stanimirov et al., 2015). FXR has been shown to be preferentially activated by unconjugated bile acids and to regulate a number of metabolic processes (Stanimirov et al., 2012). FXR regulates the bile acid homeostasis by several mechanisms, including the expression modulation of uptake and efflux transporters for bile acids in the membranes of hepatocytes (NTCP and BSEP) and enterocytes (ASBT and OSTα/OSTβ). Although being the dedicated bile acid receptor, FXR may alter the expression of several uptake and efflux transporters of xenobiotic compounds. FXR influence the expression of hepatic OATP1B1 and OATP1B3 uptake transport proteins whose substrates include drugs such as statins, enalapril, methotrexate, paclitaxel, etc. Besides, this nuclear receptor regulates the expression of MRP2 and MRP3 efflux transporters in the membranes of hepatocytes and enterocytes, whose substrates include anthracyclines, HIV protease inhibitors, methotrexate, etoposide, etc. Therefore, bile acids that activate FXR can significantly impact bioavailability and pharmacokinetic properties of a number of drug molecules (Staudinger et al., 2013).

The modulating potential of bile acids for drug transporters has been extensively studied in experimental animals and humans. However, the results of *in silico* study demonstrated that different bile acids have high binding affinities toward multidrug transporters in intestinal bacteria as well, which may also contribute to altered drug pharmacokinetics (Djanic et al., 2016).

The underlying mechanisms for drug transport enhancement across different biological membranes, following the specific routes of drug administration, have also been investigated. Sodium-GCA improved the absorption of insulin when administered both rectally and *via* the lung. The proposed mechanism of action involves the increase of membrane permeability and inhibition of the proteolytic enzymes at the absorption site (Fukuda et al., 1995; Mikov and Fawcett, 2006). The major obstacle for drug transportation through skin is stratum corneum, the outer layer of skin. Bile salts can enhance the penetration of compounds into stratum corneum, followed by interaction with keratin filaments that leads to corneocytes disruption. They increase the paracellular transport by interactions with hemidesmosomes (Benson, 2005). Bile salts can induce the reversible opening of the blood–brain barrier and drug permeation enhancement. Part of the effect is hypothesized to be mediated by tight junction modulation, cell lysis or incorporation of bile salts into the lipid bilayer (Lalić-Popović et al., 2013).

## The Influence of Bile Acids Co-Administration on Drug Transport

The role of bile in digestion and absorption of lipophilic compounds is well established and many *in vitro* and *in vivo* studies confirming its significance have been described in the literature over the past decades. The oral bioavailability of the immunosuppressive drug cyclosporine A is generally low due to its high molecular weight, poor aqueous solubility, Pgp-mediated efflux from the enterocytes and extensive pre-systemic metabolism. However, it was ascertained that the bioavailability of this drug is more than threefold reduced in bile duct-cannulated rats in comparison to intact rats, when the suspension of cyclosporine A was perorally administered. The results of this study confirm the importance of bile for the solubilization of poorly soluble compounds and their subsequent absorption (Miyake et al., 1999). More recently, antimalarial drug halofantrine dissolved in PEG400 has been administered to sham-operated and bile duct-cannulated rats, and the higher rate and extent to which a drug is absorbed has been determined in animals with the bile present in the intestinal lumen. It was demonstrated that bile could prevent the precipitation or facilitate a dissolution of the precipitate, which is very important for drug formulations with a high precipitation potential, such as systems with high co-solvent concentrations (Tønsberg et al., 2011).

Bile salts, as the main constituents of bile, have been intensively investigated for their absorption-enhancing effects when co-administered with various drugs. The co-administration of bile salts proved to increase the bioavailability of cyclosporine A in both rats and humans. While tauro-ursodeoxycholate (TUDCA) managed to significantly increase the absorption of cyclosporine A from the self-emulsifying delivery system (Sandimmune^®^) in rats (Balandraud-Pieri et al., 1997), the supplementation with 400 mg of CA and 100 mg of semisynthetic dehydrocholic acid (3,7,12-triketocholic acid) improved cyclosporine A bioavailability in healthy volunteers and in kidney transplant patients (Lindholm et al., 1990).

The addition of bile salts sodium-GCA and sodium-DCA enhanced bioavailability of hypolipidemic drug lovastatin 5- and 11-fold, respectively, following the oral administration to rats. The higher area under the concentration-time curve (AUC) values by the addition of bile salts, reflecting improved oral bioavailability, can be attributed to the increased solubility of lovastatin, since the poor aqueous solubility is the limiting factor for its absorption. Besides, the enhanced bioavailability can also be a consequence of the Pgp transporter inhibition, since lovastatin is known to be a both Pgp substrate and inhibitor (Kim et al., 2011).

Due to high toxicity of some hydrophobic bile acids, various semisynthetic bile acid analogs with improved toxicological and pharmacokinetic properties have been developed. Cholylsarcosine is a non-toxic bile acid derivative that has been investigated as absorption enhancer. This semisynthetic bile acid increased *in vitro* and *in vivo* permeation and absorption of the peptide drugs, octreotide and desmopressin, although to a lower extent in a comparison to CDCA. However, cholylsarcosine can be potentially used for absorption enhancement of other peptide drugs, such as calcitonin and parathyroid hormone, due to its favorable toxicological profile (Michael et al., 2000).

Much attention has been paid to pharmacological studies of semisynthetic bile acid keto-derivatives as absorption enhancers, primarily 12-monoketocholic acid (MKC), considering their diminished membranolytic activity and preserved absorption-promoting properties. Ampicillin is amphoteric and sparingly soluble antibiotic drug in water, whose oral bioavailability is less than 50%. The co-administration with MKC resulted in the significant increase of the ampicillin maximum plasma concentration (*C*_max_) and the AUC value in rats, which indicates that a concomitant use of ampicillin with MKC could improve therapeutic efficacy of ampicillin and extend its clinical use (Mikov et al., 2005).

Similarly, the bioavailability of antidiabetic drug gliclazide was significantly increased in healthy rats when co-administered with MKC, affecting no differences in glucose levels. On the other hand, gliclazide bioavailability was much lower in diabetic rats and was not altered by co-administration with MKC (Mikov et al., 2008). Given that another sulphonylurea derivative glibenclamide act as an inhibitor of various ATP-binding cassette (ABC) transporters, including multidrug resistance efflux pumps (Payen et al., 2001; Tournier et al., 2013), the role of these efflux proteins in gliclazide transcellular transport, alone and in combination with MKC, was investigated in healthy and diabetic rats. It was reported that MKC reduced the mucosal to serosal absorption in healthy rats, which may be the result of the selective inhibition of Mrp3 transporters. On the contrary, MKC didn’t induce any net flux of gliclazide in diabetic rats, probably due to lack of action of drug transporters involved or the suppression of their expression (Al-Salami et al., 2008).

In experiments with methotrexate, the hydrophilic drug whose absorption is limited by its low membrane permeability, it was determined that sodium salts of CA and MKC decreased the apical to basolateral permeation of methotrexate across Caco-2 cells at low concentrations (0.25–1 mM) and increased it at higher concentrations (3–5 mM). Membrane integrity was shown to be disrupted at higher concentrations of bile salts. In contrast, the pharmacokinetic study in rats showed that MKC at 4 and 20 mg/kg did not change the bioavailability of methotrexate, whereas at higher doses of 40 and 80 mg/kg significantly reduced it (Chen G. et al., 2009). Accordingly, several keto-derivatives of CA, including MKC, were shown to decrease methotrexate uptake in Caco-2 cells despite increasing membrane fluidity. Therefore, it was suggested that ketocholates inhibit methotrexate uptake transporters, probably indirectly through the disturbance of their lipid environment (Chen et al., 2012).

The inhibitory effects of bile salts on drug absorption was observed in several other studies. The absorption kinetics of poorly water-soluble antifungal drugs ketoconazole and griseofulvin from the small intestine was studied *in situ* in the intestinal loop of the rat. The addition of concentrations of sodium-TCA above the CMC in the perfusion solution resulted in a reduction of the absorption rate of both studied lipophilic drugs. This reduction in the absorption kinetics may be a consequence of the decrease of the free drug fraction in solution due to micellar solubilization (Poelma et al., 1990). It was also demonstrated in humans that the co-administration of bile acids can reduce the drug absorption. Unconjugated bile acids CDCA and UDCA decreased the oral bioavailability of the lipophilic drug nitrendipine, which can be explained by relatively low aqueous solubility of unconjugated bile acids and their potential to reduce the solubilization capacity of the existing bile salt micelles formed by endogenous bile (Sasaki et al., 2001).

Besides exerting absorption-enhancing effects following the peroral administration of drugs, bile acids can modulate the transport of drugs when administered *via* other routes as well. The buccal route can be advantageous for drugs when a rapid onset of action is required. Therefore, the influence of sodium-GCA on the transport of antiarrhythmic agents, sotalol and flecainide, across porcine buccal mucosa was studied. Sodium-GCA seemed to be an effective penetration enhancer for the buccal absorption of the more polar ionized form of flecainide in an aqueous solution, while it could not improve the transport of sotalol, probably due to ion-pairing (Deneer et al., 2002).

Over the years, many attempts have been made to exploit the alternative routes of insulin delivery. Considering the enzymatic and penetration barriers to peptide and protein absorption from the gastrointestinal tract, many researchers have investigated bile acids as insulin absorption enhancers for nasal and pulmonary delivery. Nasal absorption-promoting activity of bile salts was determined for insulin in both experimental animals and human subjects. In the clinical study, the hydrophobicity of unconjugated bile salts correlated positively with nasal absorption of insulin, and the most hydrophobic bile salt, sodium-DCA, produced the most pronounced elevation in serum insulin level and reduction in blood glucose concentration (Gordon et al., 1985). Besides natural bile acids, the semisynthetic MKC also managed to significantly improve nasal absorption of insulin in rats (Kuhajda et al., 1997). The co-administration of sodium-TCA strongly increased the bioavailability of nebulized insulin in dogs, and the possible explanation for improved pulmonary absorption includes the modulation of the aggregation state of insulin and production of insulin monomers, as well as widening tight junctions between airway epithelial cells (Johansson et al., 2002).

Insulin was also explored with respect to rectal absorption and it was demonstrated in rabbits that insulin could cross the rectal mucosa only after the addition of penetration enhancers, predominantly after the co-administration with sodium-DCA (Yamamoto et al., 1992). Besides, rectal administration of insulin with 5% sodium-GCA produced a strong hypoglycemic effect in rats, approximately half as effectively as intramuscular insulin in the presence of this bile salt (Aungst et al., 1988).

Bile salts were tested as ocular penetration enhancers for β-blocking agents, differing in their polarity, through isolated rabbit corneas. Generally, they increased the permeation rates of hydrophilic drugs, atenolol and timolol, more efficiently than those of hydrophobic drugs, levobunolol and betaxolol. TDCA in a concentration of 0.05% produced the most pronounced absorption-enhancing effect without increasing the corneal hydration level beyond the safety level and with no irritant activity observed *in vivo* at this concentration (Saettone et al., 1996).

Skin permeation of drugs can also be enhanced by co-administration with bile acids. There has been an increasing interest toward delivery of non-steroidal anti-inflammatory drugs (NSAIDs) transdermally, to avoid the problems associated with their oral administration. Thus, the addition of sodium-GCA significantly improved the permeation of the tromethamine salt of NSAID ketorolac across the rat skin (Fetih et al., 2011). Besides, bile salts sodium tauroglycocholate and sodium-DCA managed to enhance *in vitro* permeation of theophylline through shed snake skin and their permeation-enhancing activity positively correlated with surface activity (Moghimipour et al., 2012). The hydrogel formulation obtained by the addition of sodium-DCA and containing corticosteroid drug betamethasone-17-valerate was tested for its *in vitro* skin permeation characteristics and *in vivo* anti-inflammatory activity. It was demonstrated that transdermal permeation of this corticosteroid across the rat skin was eightfold elevated in comparison to the commercial cream with the same concentration of the active substance. Furthermore, *in vivo* anti-inflammatory activity was in agreement with *in vitro* drug permeation (Senyiit et al., 2011).

Keto derivative of CA, MKC, was investigated also as a potential permeation enhancer through the blood–brain barrier. It was first determined that MKC induced the increase of quinine uptake up to twofold into the central nervous system in rats. In the test of quinine uptake, methyl ester of MKC did not show a promoting effect, which can suggest its specific action. Besides, MKC enhanced the analgesic activity of morphine and the hypnotic activity of pentobarbital. In animals pretreated with MKC, pentobarbital-induced sleep induction time was significantly shorter and the sleeping time was considerably longer in comparison to the control group (Mikov et al., 2004). On the other hand, MKC caused no change in the analgesic activity of tramadol in rats. The promoting effect of MKC on the analgesic action of morphine can be explained by the formation of a hydrophilic complex, which is transferred more easily through the blood–brain barrier compared to the morphine molecule alone (Kuhajda et al., 2009). In addition to MKC, natural bile acid DCA was also shown to enhance the permeability of antidiabetic drug gliclazide through the blood–brain barrier in healthy and diabetic rats. Probable mechanism of blood–brain barrier opening is the modification of tight junctions or incorporation of bile salt in the membrane bilayer, while cell lysis and brain edema can be excluded since concentration of DCA at the site of action was lower than 1.5 mM and brain edemas were not observed in this study (Lalić-Popović et al., 2013).

Recently, the absorption enhancer sodium-DCA has been demonstrated to promote high gene transfer in skeletal muscles and this bile salt-mediated muscle gene transfer might have broad applications in gene therapy. Gene delivery to skeletal muscles is a promising strategy for the treatment of muscle disorders and for the systemic secretion of therapeutic proteins. Sodium-DCA increased more than 100-fold the levels of the reporter gene luciferase compared to naked DNA after intramuscular injection. The results indicated that sodium-DCA needs to be injected first, suggesting that bile salt permeabilizes membranes for at least 2 h, which allows DNA to penetrate into the muscle cells (Leborgne et al., 2017).

## Physical Complexation and Chemical Conjugation of Bile Acids With Drug Molecules

Besides micellar solubilization, there are many other types of interactions between bile acids and drug molecules, which can influence the drug transport across the biological membranes. Most common drug-bile salt interaction is ion-pairing and the formed complexes may have either higher or lower polarity compared to the drug molecule itself. Furthermore, the hydroxyl and carboxyl groups of bile acids can be utilized for the covalent conjugation of drugs, which changes their physicochemical and pharmacokinetic properties.

The chemical conjugation of drugs with bile acids may contribute to their improved bioavailability following the oral administration due to ability of bile acids to withstand the gastric and enzymatic degradations in the stomach or to promote the active absorption *via* ASBT in the ileum. Regarding oral delivery formulations, the bile acid is conjugated through the covalent amide bonding in most cases, although the bile acid selection and conjugation method should be compatible with the desired form of the formulation and the route of administration (Nurunnabi et al., 2016). Bile acid-drug chemical conjugates represent novel delivery systems for drugs, which will be more in-depth discussed in the following section (see section “Bile Acid-Drug Conjugates”).

Different strategies for chemical conjugation of bile acids with carriers have been developed as well. Thus, LCA-derived phospholipid was synthesized in order to enhance the absorption and pharmacological effects of conjugated drugs. The conjugation of a lipophilic anticancer drug tamoxifen to the natural bile acid LCA using amide linkage provided the gastric pH stability, while the introduction of phosphocholine head group at 3′-hydroxy terminal of LCA aided in the formation of mixed micelles in the intestine and facilitated the absorption of tamoxifen. Pharmacokinetic and biodistribution studies in 4T1 tumor bearing mice confirmed the enhanced intestinal absorption and the accumulation of phospholipid-drug conjugate in the tumor, leading to improved anticancer activity of tamoxifen (Sreekanth et al., 2017).

At physiological pH, bile acids as weak acids are anionic in aqueous solutions and may directly interact with basic (cationic) drug molecules in the intestinal lumen and thereby impact their aqueous/lipid transition. The potential of bile acids to alter the partitioning of ionized drugs has been demonstrated for the amine drug quinine. The absorption of quinine in rabbits was significantly reduced when co-administered with sodium-GDCA, which can be attributed to the formation of an ion-pair complex between the anionic bile salt and the cationic quinine (Dongowski et al., 2005). The formation of more hydrophobic ion complexes with bile salts has been suggested also for trospium chloride, a quaternary amine with high aqueous solubility and low membrane permeability. The apparent partition coefficient of trospium chloride into a lipophilic octanol phase was increased in the presence of bile salts, and both the structure and concentration of bile salts influenced the ion-pairing and distribution. Accordingly, the formation of this complex enhanced the absorption of trospium chloride across Caco-2 cells and excised rat jejunum (Heinen et al., 2013). Bile salts also facilitated the distribution of cationic β-blocking drug propranolol into liposomes, but it was most likely caused by the insertion of bile salts into the liposomal membrane, leading to increased electrostatic interactions. The formation of complexes between positively charged propranolol molecules and bile salts in the aqueous phase was shown to have a minor influence on distribution. The studied bile salts increased the distribution in a concentration- and bile salt-specific manner, with DCA exerting the most prominent effect (Yang et al., 2011).

The polar antibiotic drugs kanamycin, amikacin, and vancomycin, have been also shown to form the hydrophobic ionic complexes with DCA and UDCA. The obtained complexes were structurally characterized and they showed higher inhibition of *Staphylococcus aureus* growth compared to parent drugs. The aminoglycoside complexes, in particular kanamycin-DCA, exhibited strong inhibition of biofilm formation as well as significant dispersion capacity on methicillin-resistant (MRSA) clinical isolates, while complexes with vancomycin were generally less effective. All investigated drugs are water-soluble with inadequate penetration into the cells. However, the aminoglycosides kanamycin and amikacin are strongly basic compounds that exist as polycations at physiological pH, while vancomycin is an amphoteric and high-molecular-weight glycopeptide. It was suggested that the application of aminoglycoside complexes with DCA as dry powders for pulmonary administration could be advantageous due to their biofilm inhibition activity that is often associated with the frequent failures of antibiotic treatments (Giovagnoli et al., 2017).

The cationic peptides, such as salmon calcitonin (sCT) and epidermal growth factor receptor-targeted hybrid peptide, can be complexed with anionic bile acids through electrostatic interactions. The addition of bile salts, primarily sodium-TDCA, significantly increased the permeability of sCT across Caco-2 cell monolayers, probably due to ion-pair forming. Besides, the administration of sodium-TDCA proliposomes resulted in a sevenfold increase in the bioavailability of sCT, when administered duodenally to rats (Song et al., 2005). Similar results were obtained for epidermal growth factor receptor-targeted hybrid peptide, in which epidermal growth factor receptor (EGFR)-binding peptide was conjugated with lytic peptide. This hybrid peptide is cationic due to presence of multiple lysine moieties. The *in vitro* permeability of the peptide complex with TDCA across Caco-2 cells was fivefold enhanced in comparison to the peptide alone. Furthermore, *in vivo* mouse xenograft model of human gastric cancer treated with peptide-bile acid complex showed a 1.6-fold reduction in the mean tumor volume as compared with the peptide alone (Gaowa et al., 2018).

In order to form hydrophobic ionic complexes with acidic drugs that would have enhanced membrane permeability, new cationic oral bile acid-based carriers such as deoxycholylethylamine (DCEA), *N*(α)-deoxycholyl-L-lysyl-methylester (DCK) and *N*(α)-deoxycholyl-L-lysyl-hydroxide (HDCK), have been developed as absorption enhancers. Given that insulin is an anionic polypeptide at physiological pH, cationic bile acid derivative DCK, consisting of a hydrophobic core of DCA and a positively charged amine group of lysine, has been developed and complexed with insulin aspart through ion-pair interaction. This complex demonstrated considerably enhanced hydrophobicity and increased permeation across Caco-2 monolayers. The effect of DCK in enhancing the insulin absorption resulted primarily from transcellular processes and also from prominent bile acid transporter activity in the ASBT-transfected MDCK cells. The pharmacokinetic profile of insulin-DCK complex delivered orally was comparable to that of insulin aspart administered subcutaneously, suggesting its possible application in the oral insulin delivery (Mahmud et al., 2015).

Similar to insulin, heparin is not absorbed orally as a result of its high molecular weight, negatively charged surface, high water solubility and degradability in an acidic stomach environment. However, studies have demonstrated that both physical complexing and chemical conjugation of heparin with bile acids could enhance its oral absorption (Nurunnabi et al., 2016). The low molecular weight heparin (LMWH) was found to physically associate with bile acid derivative DCEA by ion-pairing interaction without altering the structure. DCEA is a positively charged derivative of DCA that managed to increase the lipophilicity of complexed LMWH and to significantly enhance its bioavailability in rats. LMWH-DCEA complex was absorbed through all parts of the small intestine of rats without causing tissue damage (Lee et al., 2007). Novel heparin-based angiogenesis inhibitors have been developed to target metastatic cancers. TCA-conjugated derivative of LMWH (LHT7) proved to be a potent, multi-targeting angiogenesis inhibitor but with poor oral bioavailability and short half-life *in vivo*. Subsequently, the chemical conjugate of LHT7 and tetramer of DCA (LHTD4) was synthesized and then physically complexed with DCK through ion-pairing in order to mask the negative charge and increase the lipophilicity of LHTD4. LHTD4-DCK complex showed significantly enhanced oral bioavailability and prolonged the mean residence time after oral administration in rats, in comparison to LHTD4 alone and LHT7-DCK complex. LHTD4-DCK complex binds with the ASBT transporters in the ileum, which promotes the transcellular absorption in the intestine (Alam et al., 2014).

Bisphosphonates are widely used drugs to treat osteoporosis. They are derived from naturally occurring pyrophosphate and are characterized by low intestinal absorption, due to their high polarity and ionization at physiological pH. Physically associated DCK significantly enhanced the apparent membrane permeability of bisphosphonates ibandronate and risedronate in a parallel artificial membrane permeability assay (PAMPA) model, in comparison to bisphosphonates applied alone. Besides, the oral bioavailability of bisphosphonates in rats was also promoted by complex formation with DCK. The physical complex of acidic bisphosphonates with DCK enhanced their hydrophobicity, which is known to be a key factor required for increasing drug permeability across the biological membranes (Park et al., 2013; Park and Byun, 2014).

Ceftriaxone is a long acting, broad-spectrum third-generation cephalosporin antibiotic for parenteral use. This drug contains two negatively ionized carboxyl groups, which are responsible for high water solubility and low absorption in the intestine. Cationic bile acid-derived drug carrier DCEA formed physical complexes at varied molar ratios with ceftriaxone. This complexation increased the partition coefficient of ceftriaxone and it was demonstrated that ceftriaxone became more hydrophobic as the molar ratio of the carrier in the complex increased. As a consequence of increased lipophilicity, ceftriaxone-DCEA complex had a significantly higher bioavailability in rats compared to the antibiotic alone (Lee et al., 2005b). Another oral drug carrier derived from DCA, HDCK, was also shown to form the stable physical complexes with ceftriaxone. HDCK increased the apparent membrane permeability of ceftriaxone in the artificial *in vitro* PAMPA model, which indicated the enhancement of passive diffusion. Furthermore, ceftriaxone-HDCK ionic complex permeated Caco-2 monolayers *via* transcellular pathway, and the interaction of this complex with ASBT transporter and subsequent active absorption were confirmed in MDCK cells transfected with *ASBT* gene. Finally, the optimized formulation containing the ceftriaxone-HDCK complex exhibited significantly increased oral bioavailability of ceftriaxone in non-human primates (Jeon et al., 2013).

The formation of hydrophobic ionic complexes between drugs and bile acids or their cationic derivatives is particularly significant for drugs belonging to the class III of the Biopharmaceutics Classification System (BCS). Those drugs possess high aqueous solubility and poor intestinal permeability and their oral bioavailability may be improved by increasing their lipophilicity or by using permeation enhancers, such as bile acids. In contrast, more hydrophilic physical complexes between drugs and bile acids can be formed through hydrophobic interactions, which can be beneficial for the drugs belonging to the class II of the BCS, whose limiting factor for absorption is low water solubility.

Simvastatin is highly lipophilic drug with extremely low water solubility that may exist in prodrug lactone and active β-hydroxy acid form. Although simvastatin has low oral bioavailability (<5%), which may be attributed to its slow dissolution rate in the gastrointestinal tract, i.e., low intestinal uptake, coupled with extensive first pass metabolism (Geboers et al., 2016), this drug has been used for years to treat hypercholesterolemia because it inhibits HMG-CoA reductase in the liver. However, recent evidence suggests that the beneficial effects of statins may not only be due to their lipid-lowering properties, but also to their cholesterol-independent or pleiotropic effects, and therefore concentrations of statins in systemic circulation need to be sufficient for exerting these effects. Several novel strategies for enhancing the solubility of simvastatin and its bioavailability have been reported, including the preparation of solid dispersions (Javeer et al., 2013). The addition of submicellar concentrations of bile salts (CA, DCA, and MKC) into the *n*-octanol/buffer system decreased the values of distribution coefficient of both simvastatin forms, which was suggested to be the consequence of the formation of hydrophilic complexes with increased solubility in aqueous phase. This could contribute to increased simvastatin absorption and bioavailability. The complexation of simvastatin with bile acids was analyzed using *in silico* computational studies and the hydrophobic interactions of simvastatin with the bile salt steroid nucleus were proposed. In the model of simvastatin-MKC complexes, MKC was predicted to bind to simvastatin by hydrophobic interactions, while the hydroxyl and keto groups were oriented toward the outer side of the aggregate, making this complex more hydrophilic than simvastatin molecule itself (Danić et al., 2016).

Similarly, the increased analgesic effect of morphine when co-administered with MKC in rats may be explained by the possible interactions based on the molecular models. Morphine has the polar region characterized by the hydroxyl groups and the ether oxygen, while the rest of the molecule is mainly hydrophobic. Morphine can interact with MKC in two ways. First, it can bind to the α-side of MKC *via* hydrogen bonds, hiding the polar part of the morphine molecule and making the complex more hydrophobic than the morphine itself. On the other hand, morphine can bind simultaneously two molecules of MKC, forming the hydrophilic aggregate, whereby the hydroxyl and keto groups are oriented toward the outer side. The promoting activity of MKC on the morphine transfer across the blood–brain barrier is probably a result of the formation of hydrophobic complexes in the membrane, which accelerates the entering of morphine in the lipid bilayer, and formation of hydrophilic aggregates in the intracellular space, facilitating the further transport of morphine (Kuhajda et al., 2009).

The significance of hydrogen bonds in the formation of bile acid-drug complexes, and thus in the permeation-enhancing activity of bile acids, has been confirmed in the study examining the influence of natural bile acids CA, CDCA, DCA and their keto derivatives on the *in vitro* transport of lidocaine and verapamil from an aqueous medium to the intestinal membrane of rats. The transport of lidocaine was significantly increased by 7-keto-DCA, whereas verapamil transport was significantly influenced by CA. Such effects are explained by chemical properties and differences in proton-donor and proton-acceptor groups of corresponding bile acids and drugs. Namely, of all the tested bile acids, 7-keto-DCA and CA form the most stable hydrogen-bonded complexes with the corresponding drug (Poša and Kuhajda, 2010).

## Pharmaceutical Formulations and Drug Delivery Systems Containing Bile Acids

Bile acids can be utilized in the formulation of conventional dosage forms, but also of novel micellar, vesicular and polymer-based therapeutic systems. Their peculiar structural properties make them suitable for the formation of supramolecular aggregates, such as micellar systems, although hyperbranched structures and hydrogels can be developed as well (Stojančević et al., 2013). The availability and low cost of bile acids, along with their simple derivatization procedures, turn them into attractive building blocks for the design of novel pharmaceutical formulations and systems for the delivery of drugs, biomolecules and vaccines. Considering the stability of bile acids in acidic stomach environment, their adaptability to dynamic pH variations and the presence of selective uptake transporters in the intestine, bile acid-based therapeutic systems may be suitable for oral drug delivery (Faustino et al., 2016; Nurunnabi et al., 2016).

### Bile Acids in Conventional Dosage Forms

Although bile acids have not been commercialized as excipients in conventional dosage forms such as tablets, probably due to higher price in comparison to commonly used tablet lubricants, they are being investigated as excipients specifically for drug molecules that need permeation enhancers to be readily absorbed. Bile acids in the tablet formulations can be exploited to enhance the release, i.e., dissolution rate of the active substance as well. Tablets containing ranitidine hydrochloride, theophylline and phenobarbital as active principles, and bile salts as excipients, have been developed. Magnesium stearate, as a widely used lubricant in tablets, was replaced with the equimolar concentrations of sodium salts of CA, MKC, and dehydrocholic acid (3,7,12-triketocholic acid) in order to determine if bile salts could act as surfactants and lubricants in tablet formulations and thus impact the drug release. Dissolution rate was increased significantly for all three drug substances, although they differ in their physicochemical properties: ranitidine hydrochloride is a hydrophilic acid salt, aminophylline is a hydrophilic weak base and phenobarbital is a lipophilic weak acid. All studied bile salts increased the dissolution rate, with keto-derivatives exerting more pronounced effects in comparison to sodium-CA (Pocuca et al., 2017).

Bile acids have been also investigated as excipients in novel tablet formulations. Buccal bioadhesive bilayer tablets of the antiemetic drug prochlorperazine maleate were designed and formulated by using buccoadhesive polymers and bile salts (sodium-DCA, sodium-GCA, and sodium-TCA) as permeation enhancers, in order to provide a rapid onset of action. The addition of bile salts in the formulations, particularly sodium-GCA, resulted in a remarkable increase in the flux of prochlorperazine maleate through porcine buccal mucosa. Sodium-GCA did not considerably change bioadhesive strength, but increased the swelling index of the tablet formulation. Additionally, *in vitro* release studies were performed and the amount of drug released after 6 h from the buccal tablets containing bile salts was significantly higher compared to the marketed conventional tablets (Jain et al., 2016).

### Mixed Micellar Systems

The bile acid-based micellar drug delivery systems use bile acids to self-assemble into the micelles and pack the hydrophobic drugs inside with a high capacity. The inclusion of the lipophilic components usually lowers the CMC value and increases the size and solubilization efficacy of the bile acid aggregates. Bile acids can form mixed micelles when combined with polar lipids, conventional surfactants or amphiphilic drugs. It was demonstrated that the combined use of bile salts with phospholipids, fatty acids or polyamines may improve their effectiveness as absorption enhancers and allow a decrease in their concentration, thus reducing the risks of toxic membranolytic effect (Miyake et al., 2006). Phospholipids such as lecithin were shown to buffer the membranolytic properties of hydrophobic bile salts such as sodium-DCA and attenuate their cytotoxic effects due to mixed micelle formation (Tan et al., 2013).

The mixed micellar systems consisting of sodium-CA at a set concentration of 40 mM, in association with different fatty acids (caprylic acid, oleic acid and linoleic acid), enhanced the absorption of a highly lipophilic drug clofazimine in rat gut perfusion studies to a greater extent in comparison to non-micellar and simple micellar systems. The maximum enhancement in both solubility and the rate of clofazimine absorption was obtained with the equimolar sodium-CA/linoleic acid system (O’Reilly et al., 1994). Similarly, sodium-TCA/linoleic acid mixed micelles significantly increased the absorption of mannitol, PEG 900 and PEG 4000 in Caco-2 cells, probably *via* both paracellular and transcellular routes, but without inducing membrane damage (Meaney and O’Driscoll, 2000).

There are several commercial bile acid-based mixed micellar formulations containing lipophilic drugs for parenteral administration, such as vitamin K1 or phytomenadion (Konakion^®^ MM), diazepam (Valium^®^ MM) and amphotericin B (Fungizone^®^). Lipophilic vitamin K1 and poor water-soluble benzodiazepine drug diazepam are solubilized in the mixed micelles consisting of GCA and soy lecithin, while the antifungal drug amphotericin B, a hydrophobic polyene antibiotic, is commercialized as micellar dispersion with sodium-DCA (Greer et al., 1998; Faustino et al., 2016). Despite its efficacy, DCA-solubilized amphotericin B is associated with severe toxic side effects, namely nephrotoxicity. Therefore, novel mixed micellar systems consisting of equimolar concentrations of bile salts (sodium-CA and sodium-DCA) and a dimeric anionic surfactant lipoamino acid were investigated as delivery systems for amphotericin B under biomimetic conditions. The gemini lipoamino acid formulations are generally reported as biocompatible, biodegradable and non-toxic, and lipoamino acid-based micelles were shown to solubilize amphotericin B in its monomeric and less toxic form and to exhibit *in vitro* antifungal activity comparable to that of the commercial DCA-based formulation (Serafim et al., 2016).

The mixed micelles composed of sodium-CA and phospholipid were tested *in vivo* as potential delivery systems for hydrophobic and poorly absorbed hepatoprotective drug silybin. The relative bioavailability of silybin was 2.5-fold higher after oral administration of silybin-loaded mixed micelles in dogs, in comparison to silybin-*N*-methylglucamine, the soluble chemical derivative of silybin (Yu et al., 2010).

The bile-acid based mixed micellar systems have been also investigated as drug carriers for hydrophilic drugs such as cefotaxime. Mixed micelles composed of phosphatidylcholine and sodium-DCA, and loaded with a cefotaxime-MKC complex, significantly increased the oral bioavailability of cefotaxime in rats, when compared to cefotaxime-MKC complex and cefotaxime alone. Cefotaxime is freely soluble in water, but has a poor oral bioavailability due to its low intestinal permeability and degradation in the acidic environment of the stomach. Thus, the suggested mechanisms for the enhancement of cefotaxime absorption include direct activity of sodium-DCA on mucosal membrane, the increased intestinal uptake by endocytosis *via* both Peyer’s patches and intestinal enterocytes, and the increased drug stability encapsulated in the mixed micelles (Arafat et al., 2017a).

### Bilosomes

**FIGURE 3:**
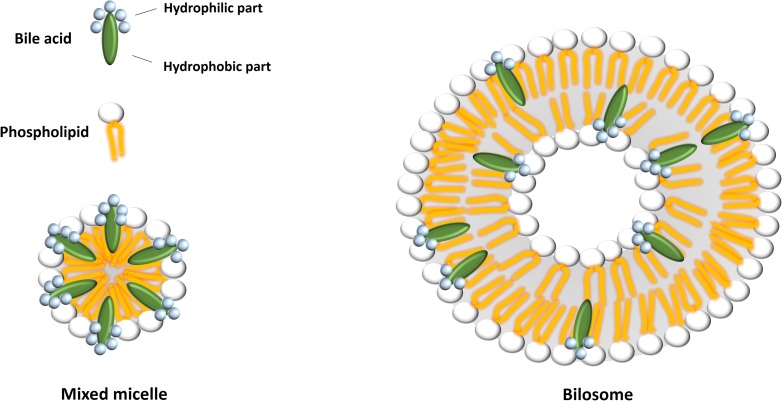
A schematic representation of mixed micelle and bilosome structures. Bilosomes possess a lipid bilayer with bile acids included, whereas mixed micelles are structures composed of a monolayer of different amphipathic molecules.

Bile salt-containing vesicles or bilosomes have been developed as delivery systems for conventional drugs and therapeutic peptides and proteins. They act as more stable drug carriers than parent liposomes and niosomes, and facilitate the transmembrane transport and absorption of drugs more efficiently. The structure of bilosome and its difference from the mixed micelle is presented at Figure 3. Several antigens, including tetanus toxoid, hepatitis B surface antigen and influenza A virus hemagglutinin, have successfully been orally delivered using bilosomes. Bilosomes represent the new hope in oral vaccine delivery, since oral administration of bilosomes loaded with antigens produce effective stimulation of both systemic and mucosal immune responses (Chilkawar et al., 2015). Although bilosomes as vaccine delivery systems have not been commercialized yet, this technology is covered by granted patents (US 5,876,721 and EP 0722341B1) and patents relating to the delivery of small molecules and biologicals are being developed (Alexander and Brewer, 1999).

Liposomes containing sodium salts of GCA, TCA, and DCA and loaded with insulin as peptide drug were developed and administered to healthy and diabetic rats. The administration of sodium-GCA bilosomes resulted in higher oral bioavailability than liposomes containing sodium-TCA or sodium-DCA and conventional liposomes, which was associated with the most pronounced hypoglycemic effect (Niu et al., 2012). The following *in vitro* and *ex vivo* stability studies confirmed that liposomes containing GCA retained significantly more encapsulated insulin in simulated gastrointestinal media in comparison to conventional liposomes, as well as in *ex vivo* gastrointestinal media from rats. These results were explained by protease-inhibiting activity of GCA, which contributed to the reduction of insulin degradation (Hu et al., 2013). These results indicate the potential use of bilosomes as delivery systems for other peptide drug molecules as well.

Bilosomes, along with liposomes and niosomes, are enclosed vesicles composed of lipid bilayers and aqueous core, and have been extensively studied as drug carriers for both hydrophilic and lipophilic drug molecules, since hydrophilic drugs can be loaded into the inner aqueous phase, whereas hydrophobic drugs can be inserted into the hydrophobic lipid bilayers. As previously described, cefotaxime is a hydrophilic third-generation cephalosporin antibiotic with low oral bioavailability. The encapsulation of this drug in liposomes containing semisynthetic bile acid MKC resulted in five and ninefold increase of cefotaxime oral bioavailability in rats, when compared to cefotaxime-loaded conventional liposomes and aqueous solution of cefotaxime, respectively (Golocorbin-Kon et al., 2009). Similarly, cefotaxime-loaded liposomes prepared with sodium-DCA showed the reduced leakage of encapsulated cefotaxime in biorelevant dissolution media. Accordingly, the oral bioavailability of cefotaxime in DCA-bilosomes was five-times higher compared to cefotaxime solution and twice as much as in conventional liposomes. The key factor for enhanced stability of DCA-bilosomes and improved intestinal permeability of the active principle was suggested to be the change in physical structural properties of vesicles, such as additional elasticity, fluidity and negative charge (Arafat et al., 2017b). These results suggest that bilosomes might extend the application of cefotaxime from parenteral only to oral application.

Sodium-DCA has been also utilized in the preparation of bilosomes containing cyclosporine A, a hydrophobic drug characterized by low aqueous solubility and low permeability and belonging to the class IV of BCS. The absorption enhancement strategies for these drugs are very challenging since their bioavailability cannot be simply improved by solubilization mechanisms. The pharmacokinetic study in rats revealed that the oral bioavailability of cyclosporine A encapsulated in DCA-bilosomes was around 20% higher in comparison to the commercially-available microemulsion-based formulation of cyclosporine A (Guan et al., 2011). Similarly, the oral bioavailability of a highly lipophilic drug fenofibrate was fivefold increased by using liposomes containing sodium-DCA compared to the fast release formulation of micronized fenofibrate. Given that DCA-bilosomes showed significantly higher oral bioavailability of fenofibrate than conventional cholesterol liposomes, it was suggested that DCA in lipid bilayers may facilitate fast transition from vesicles to mixed micelles. Besides, the ultradeformability of bilosomes may allow carrier-mediated transmembrane absorption, which can also enhance the bioavailability of fenofibrate (Chen Y. et al., 2009).

Bilosomes prepared from non-ionic sorbitan alkyl esters and different concentrations of sodium-CA and sodium-TCA provided significant enhancement of intestinal absorption of the liposoluble β-blocker carvedilol after oral administration to rats in comparison to the drug suspension and plain niosomes. This study revealed that the type and concentration of bile salts had a considerable impact on oral bioavailability of carvedilol. The administration of bilosomes caused no signs of inflammation or damage in the intestine (Arzani et al., 2015). Bilosomes composed of various ratios of phosphatidylcholine and sodium-DCA, and loaded with eprosartan mesylate as the active substance, have been prepared and characterized. The optimized formulation of this lipophilic angiotensin II receptor antagonist exerted nephroprotective effects in streptozotocin-induced diabetes rat model, as demonstrated by the substantial changes in biochemical markers of renal function and histopathological changes as well (Ahad et al., 2018).

Although initially developed for oral delivery, bilosomes have recently been evaluated for the drug delivery *via* other routes. Bilosomes consisting of sorbitan esters as non-ionic surfactants, cholesterol and sodium-DCA and loaded with a long-acting NSAID tenoxicam were investigated as carrier systems for transdermal delivery. The *ex vivo* skin permeation and *in vivo* skin deposition studies indicated that bilosomes penetrated deep within the skin and promoted permeation and deposition of tenoxicam in the skin, which is an essential prerequisite for effective transdermal delivery (Al-Mahallawi et al., 2015). Similarly, the niosomes containing the bile salt sodium-DCA were formulated with melatonin for intranasal application, which could be used to improve sleep quality or synchronize biological rhythms due to jet lag. The addition of sodium-DCA reduced the vesicle sizes, enhanced the physical stability and improved melatonin encapsulation efficiency into the niosomes, with no additional effect on cytotoxicity when compared to melatonin-loaded plain niosomes (Priprem et al., 2017). Furthermore, bilosomes for ocular delivery of poorly soluble drugs tacrolimus and terconazole have been developed. Tacrolimus is used to inhibit immunological rejection after corneal transplantation, but has difficulty in penetrating the corneal epithelium mainly due to its low aqueous solubility. The *ex vivo* corneal transport experiments indicated that bilosomes enhance the permeation of tacrolimus across the cornea up to fourfold in comparison to conventional liposomes. Bilosomes composed of sodium-TCA and sodium-GCA were well tolerated, whereas those containing sodium-DCA were toxic to human corneal epithelial cells and rabbit cornea (Dai et al., 2013). Bilosomes loaded with the antifungal drug terconazole were prepared using sorbitan esters, cholesterol, and sodium-TCA, and the edge activators (macrogolglycerol ricinoleate and macrogolglycerol hydroxystearate) that impart extra elasticity to the vesicles were added in order to obtain ultradeformable bilosomes. The optimized formulation of ultradeformable bilosomes exhibited superior *ex vivo* drug flux through rabbit cornea when compared to conventional bilosomes, niosomes, and drug suspension (Abdelbary et al., 2016). The presented data indicate the potential of bilosomes to be exploited as therapeutic systems delivered by routes other than oral administration, which may be beneficial especially for drugs unstable in the gastrointestinal tract.

### Bile Acid-Polymer Nanocarriers

Bile acid-polymer hybrid nanosystems have gained considerable attention in the field of the novel drug delivery systems development, especially for the antitumor therapy. The aim of covalent modifications of polymers using bile acids is the creation of new amphiphilic nanosystems capable of self-aggregation as a result of the hydrophobic interactions between bile acid moieties. According to the position of the reactive groups along the polymer backbone, polymers with pendant bile acid moieties or polymers end-capped with bile acids can be obtained. These hybrid amphiphiles have been widely tested as potential drug delivery systems, either in the form of physical mixtures with drugs, or as drug-polymer conjugates (Zhu and Nichifor, 2002).

Bile acid-conjugated polymers form nano-sized micelles in aqueous environments, characterized by a unique core-shell structure with tunable size and drug-loading capacity. These nanocarriers can be optimized for both passive and active targeting of anticancer drugs through the adjustment of their size to favor the enhanced permeability and retention (EPR) effect in tumor vasculature and through the surface engineering for selective targeting of key tumor receptors. Besides, the stimuli-responsive components can be introduced in bile acid-polymer nanocarriers, making them convenient for the design of smart drug delivery systems (Faustino et al., 2016).

Different synthetic polymers, such as polyethylene glycol (PEG), polyethylene imine, poloxamer 407, poly(lactic-co-glycolic acid) (PLGA), and poly-ε-caprolactone (PCL), and biological polymers, such as chitosan, chondroitin sulfate A (CSA) and hyaluronic acid (HA), have been conjugated with bile acids to generate micelle-mediated drug delivery systems. Several types of bile acid-based polymeric micellar systems have been developed, including micelles composed of PEGylated bile acids, telodendrimer-based nanomicelles and star-shaped polymeric micelles (Nurunnabi et al., 2016). The peculiar chemical structure of bile acids makes them suitable for adjusting the drug release from polymeric micelles. It has been demonstrated that the addition of DCA to the micellar solution of the block copolymer, methoxy polyethylene glycol-poly(D,L-lactic acid) (mPEG-PDLLA), alter the molecular geometry of the core-shell nanostructure. At low concentrations of DCA, face-to-face bile acid dimers could form and insert into the core of micelles, which could accelerate the drug release. On the other hand, at higher DCA concentrations, a new back-to-back DCA dimer could form in the PEG shell region, which could reduce the rate of drug release. These results demonstrate the future potential of bile acids in polymeric micellar systems for controlled drug delivery applications (Tan et al., 2018).

PEGylated bile acids can self-associate to make polymeric micelles and used for preparing self-emulsifying drug delivery systems (SEDDSs) for hydrophobic drugs. PEGylated CA, DCA, and LCA were synthesized and mixed with oleic acid to prepare SEDD for itraconazole, varying the number and length of PEG arms to tune the hydrophilic–lipophilic balance for optimizing the drug loading efficiency and biocompatibility of the corresponding formulations. The solubility of itraconazole was shown to be significantly improved in this system, along with high capacity loading, leading to a better bioavailability in rats compared with itraconazole itself and commercially available formulations. Furthermore, it was concluded that SEDDs based on BA-PEGs composed of short PEG chains give promise to an efficient oral delivery system, while those with longer PEG chains are good candidates for parenteral carriers providing improved bioavailability of itraconazole (Le Dévédec et al., 2013). Similarly, CA-polyethylenimine conjugate was synthesized and folic acid was further attached to the polymeric micelles in order to assist their internalization in cancer cells, due to high expression of folate receptor in numerous tumors. This polymeric conjugate exhibited a low CMC, small average particle size, high stability and high entrapment efficiency for doxorubicin and for siRNA, indicating the potential of this nanoconjugate to achieve targeted co-delivery of drugs and siRNA (Amjad et al., 2015).

Taxane-based chemotherapy is one of the most widely used therapies for cancer treatment. Currently, three members of taxane family, namely paclitaxel, docetaxel, and cabazitaxel have been approved for clinical use. Despite their widespread popularity, all three taxanes are poorly water-soluble, thereby making development of effective formulations for medicinal use challenging, with bile acid-based polymer nanosystems drawing special attention in this field. The micellar formulation consisting of sodium-CA and the polymer mPEG-PDLLA that is approved pharmaceutical excipient, exerted tumor-targeted delivery of paclitaxel and enhanced the drug penetration in tumor. Pharmacokinetic study in rats revealed that AUC value of these CA-based polymeric micelles was 1.8-fold higher than that of polymeric micelles without CA, and 5.2-fold higher than that of Taxol^®^, a clinically available paclitaxel formulation. The improved anticancer efficiency of this CA-polymer micellar system has been determined in BEL7402 and A549 cell-bearing nude mice, and the underlying mechanisms were suggested including the size reduction and improved stability of micelles by adding CA, which make them favorable for passive targeting to the tumor. Besides, CA enhanced the circulation time of micelles in the bloodstream, but also affected the transmembrane permeability and thus enhanced the cellular uptake, which both favored the accumulation of micelles in tumors (Zhang et al., 2017).

The linear-dendritic block copolymers, named telodendrimers, are hybrid systems which incorporate linear and hyperbranched architectures offering novel nanostructures for drugs, either covalently bound or physically entrapped. Paclitaxel has been incorporated in telodendrimer-based micelles composed of PEG with a molecular weight of 5000 Da, CA and lysine. The representative PEG^5K^-CA_8_ telodendrimer solubilized paclitaxel with high loading capacity and exhibited the similar *in vitro* cytotoxic activity against ovarian cancer cells as Taxol^®^ or paclitaxel/human serum albumin nanoaggregate (Abraxane^®^). It was well tolerated *in vivo* and achieved superior antitumor effects compared to Taxol^®^ and Abraxane^®^ at equivalent doses of paclitaxel in murine models of ovarian cancer, which can be the result of its preferential tumor accumulation (Xiao et al., 2009). Further, the hybrid telodendrimers were generated by replacing four of the eight CAs with biocompatible organic moieties, including fatty acids, cyclic vitamins, a flavoring agent and food preservative, and they were tested for their ability to encapsulate all three taxane drugs. Loading efficiency was nearly 100% when the initial amount of docetaxel and cabazitaxel used was less than 20 wt% of the polymer. PEG^5K^-(Cinnamic acid)_4_/CA_4_ seemed to be the best telodendrimer of the lot as it stably encapsulated all members of taxane family with significant loading capacity. However, it was indicated that CA, compared with other organic moieties, has a unique physicochemical property such that its presence at 50% level at the dendron is able to maintain the stability, monodisperse property and small size of the nanocarrier (<50 nm), and to stably encapsulate hydrophobic drugs (Bharadwaj et al., 2017).

Bile acids have also been used as initiators for ring-opening polymerization and the subsequent formation of star-shaped polymeric micelles. The system of nanoparticles of star-shaped CA-core polylactide-d-α-tocopheryl PEG 1000 succinate (CA-PLA-TPGS) block copolymer was developed for paclitaxel delivery for breast cancer treatment. PLA and PLGA polymers are extensively investigated due to their biocompatibility and biodegradability, but the acidic degradation products of these polyesters can cause unfavorable effects and the degradation rate is too slow due to their hydrophobic nature. These drawbacks could be overcome by the introduction of TPGS, a water-soluble derivative of the natural form of d-α-tocopherol, into the hydrophobic PLA backbone. The star-shaped CA-PLA-TPGS nanoparticles with three branch arms were shown to achieve higher drug loading content and entrapment efficiency, resulting in faster drug release as well as higher cellular uptake and cytotoxicity than the paclitaxel-loaded PLGA nanoparticles and the linear PLA-TPGS nanoparticles. These nanoparticles also demonstrated significantly superior antitumor activity in mice than the linear PLA-TPGS nanoparticle formulation and the clinical formulation Taxol^®^ (Tang et al., 2013).

Bile acids have been also utilized in the design of novel smart drug delivery systems characterized by stimulus-responsive drug release. Redox-sensitive micelles based on hyaluronic acid-DCA (HA-DCA) conjugates containing cystamin as bioreducible linkages were developed for targeted intracellular delivery of paclitaxel. These micelles exhibited excellent drug-loading capacities for paclitaxel, good stability at physiological conditions and rapid drug release under reducing conditions. Paclitaxel-loaded HA-DCA nanomicelles can be selectively taken up to tumor cells *via* HA-receptors mediated endocytosis and then achieve rapid disassembly once internalized due to highly reducing environment of tumor cells, thereby improving intracellular drug release and increasing the antitumor efficacy (Li et al., 2012). Furthermore, thermosensitive and mucoadhesive docetaxel-loaded nanomicelles composed of poloxamer 407, poloxamer 188, Tween 80 and sodium-TCA for rectal administration have been designed. Poloxamer was selected for the formulation due to its unique thermosensitive and reverse gelation properties, since poloxamer remains in liquid state at room temperature, while gelifying at physiological body temperature. These nanomicelles possessed sufficient viscoelastic properties to remain in the upper part of the rectum for the specified time. They enhanced the absorption of docetaxel across the rectal mucosa and improved its half-life and plasma concentration, resulting in a significant *in vivo* anti-tumor efficacy (Kim et al., 2014).

### Bile Acid-Containing Microcapsules

The microencapsulation process involves the coating of a drug substance in a suitable material to protect the active ingredient from the external environment, mask undesired flavors or control release characteristics of the drug encapsulated inside the microcapsules. It is commonly used to optimize the delivery of poorly absorbed drugs especially those with high degradation rate in the intestine. Coating polymers in the microencapsulation process include natural and synthetic polymers, such as polysaccharides sodium alginate and chitosan. Alginate and chitosan are non-toxic, non-irritant, biocompatible and biodegradable, and thus widely used for targeted delivery of oral drugs, as both degrade and release drugs at alkaline pH, which makes them suitable for targeted drug delivery in the lower part of the gastrointestinal tract (Ma, 2014; Lopes et al., 2017).

Utilization of bile acids in drug microencapsulation and delivery has recently shown promise in the oral targeted delivery of antidiabetic drugs, with alginate-based microcapsules exerting well-controlled and consistent release of these drugs at the absorption site in the intestine (Woodhams and Al-Salami, 2017). However, it should be emphasized that release mechanisms of drugs from hydrophilic matrices depend on various factors such as the encapsulating polymer material (structure, molecular weight, particle size, viscosity), the encapsulated drug substance (molecular weight, solubility, particle size, dose), the release conditions (dissolution medium, pH, ionic strength, temperature) and the formulation factors (manufacturing process, geometry of the matrix) (Maderuelo et al., 2011). The swelling and release studies of microcapsules loaded with coriander essential oil, prepared by spray drying method, using alginate, chitosan, chitosan/alginate and chitosan/inulin as wall materials, revealed that microcapsules could exhibit a slow release of essential oil due to the resistance to pH and temperature variations. The swelling studies proved that the penetration of water inside the microcapsule was influenced by the type of the encapsulating material and also by medium conditions. Besides, kinetic studies demonstrated that the release of coriander essential oil from chitosan and chitosan/alginate microcapsules is a diffusion-controlled release process caused by the poor swelling degree and by the presence of oil drops at the surface or in the exterior layer of microcapsules, while the release from alginate and chitosan/inulin microcapsules is a diffusion/swelling-controlled process (Dima et al., 2016).

The new formulation of gliclazide-loaded alginate-based microcapsules containing DCA displayed appropriate excipient compatibility and structural morphology with thixotropic-pseudoplastic behavior. Besides, appropriate stability, release profile, drug-content and production yield and pH-targeted delivery at various pH values and temperatures have been confirmed as well, suggesting the potential role of this formulation in the treatment of type 2 diabetes. The addition of DCA could contribute to the optimization of the formulation through the reduction of the bead swelling of microcapsules. Furthermore, DCA could play a crucial role in enhancing gliclazide absorption in the ileum, which would help to further increase its antidiabetic effect (Mooranian et al., 2014a, 2015a).

Similarly, the incorporation of bile acids DCA and CDCA within probucol-loaded microcapsules resulted in their good morphology and stability, and may be suitable for optimized oral delivery of probucol in type 2 diabetes. The addition of CDCA resulted in more controlled release, suggesting that CDCA provided enhanced resistance to mechanical strength as well as osmotic-induced swelling. Such release may optimize probucol-targeted delivery at the lower part of the intestine, where most of its absorption is expected to take place and thereby optimizing its efficacy (Mooranian et al., 2014b, 2015b).

Bile acids have been also shown to interact with chitosan, another coating polymer for microcapsules. The sorptive properties of chitosan are attributed to the creation of a viscous polysaccharide solution in a lipid environment and the presence of an amine group in the structure, facilitating the appearance of electrostatic forces between the polymer and anionic substances, such as fatty acids and bile acids (Karolewicz, 2016). Nanostructured capsules consisting of the cationic polymer chitosan and the anionic bile salt sodium-TDCA were prepared as a result of hydrophobic and electrostatic interactions between the oppositely charged components. The formation of liquid-crystalline phases in mixtures of oppositely charged surfactant-polymer systems has been already known, but it was shown that the release rate of the model hydrophilic drug, rhodamine B, from the lamellar phase was significantly influenced by temperature and salt concentration. The results suggested that modulating the drug release from bile salt-chitosan capsules could be readily achieved and that these liquid-crystalline systems could function as stimuli-responsive or sustained-release drug delivery systems (Tangso et al., 2014).

### Bile Acid-Drug Conjugates

As previously stated, the chemical conjugation of drugs with bile acids may contribute to their improved bioavailability following the oral administration, mainly through the absorption of the conjugate *via* ASBT transporter in the ileum. However, the ileal expression of ASBT commands that a bile acid-drug conjugate must be stable to hydrolysis to reach the ASBT intact. The bile acids can be used as vehicles for both conventional drugs and peptides and proteins. These drug molecules can be coupled either to a hydroxyl group at positions C-3, C-7, or C-12 of the bile acid or to the carboxyl group at position C-24, and in most initial studies drugs have been attached to the bile acid side chain, due to the relatively simple synthesis process. The nature of the linker can be used to modulate the drug release profile. It has been demonstrated that the best molecular recognition by hepatic and ileal bile acid transporters was achieved with the conjugates having attached the drug at position C-3 (Kramer, 2011).

The conjugation of therapeutic peptides and proteins to bile acids have been considered very useful for the improvement of the intestinal absorption and systemic bioavailability of the macromolecules as well. In order to develop orally active peptide drugs, a series of 15 small linear model peptides up to a chain length of 10 amino acids were covalently coupled to the C-3 position of modified bile acid and these conjugates inhibited sodium-dependent [^3^H]taurocholate uptake by rabbit ileum brush-border membrane vesicles in a concentration-dependent manner. Besides, the affinity of these peptide-bile acid conjugates to the ASBT transporter in ileum decreased with the increase in the chain length of the model peptides (Kramer et al., 1994). Additionally, covalent coupling of a peptide to a ligand which is transported carrier-mediated, such as bile acids, may improve the peptide stability as well. It was demonstrated that, when covalently attached to DCA, recombinant human insulin can be potentially used for oral delivery, since it retained high binding affinity to the insulin receptor, but also showed prolonged biological activity in physiological conditions (Lee et al., 2005a).

LMWH-DCA conjugate is one of the first examples demonstrating that the coupling with bile acids can improve pharmacokinetic properties of drugs (Sang et al., 2005). Several further studies have indicated the importance of ASBT for the transport of heparin-bile acid conjugates. The conjugation of the oligomeric form of DCA, tetra-DCA, to LMWH exhibited 50-fold higher binding affinity to ASBT transporters and oral absorption compared with mono-DCA conjugates (Al-Hilal et al., 2014).

Five conjugates of gabapentin with CDCA were synthesized and varied in ionic nature and the presence or absence of glutamic acid linker between the bile acid and drug. Gabapentin is a zwitterionic drug that exhibits low and variable oral absorption at therapeutic doses. Among two neutral, two monoanionic, and one dianionic conjugates, the monoanionic compounds were shown to be the potent substrates of human ASBT. The conjugate with CDCA, coupled at position C-24, had a high affinity toward the ASBT transporter in the terminal ileum, suggesting its role as a potential prodrug that may increase gabapentin absorption (Rais et al., 2011). Furthermore, four prodrugs of acyclovir were synthesized, where acyclovir was conjugated to a bile acid (CA, DCA, CDCA, and UDCA) *via* a valine linker at position C-24. The prodrug acyclovir-valyl-CDCA yielded the highest affinity for human ASBT. Further characterization showed that acyclovir was catalytically liberated from this prodrug by esterase activity. The CDCA-conjugated acyclovir prodrug also exhibited an almost 12-fold enhanced passive permeability, relative to the passive permeability of acyclovir itself. Oral administration of acyclovir-valyl-CDCA to rats resulted in a twofold increase in the bioavailability of acyclovir, compared to the parent drug alone (Tolle-Sander et al., 2004).

Chemical conjugates of different drugs with bile acids are being investigated intensively, not only for their ability to target bile acid transporters in order to improve membrane permeation and oral bioavailability of drugs, but also to achieve organ-specific drug action, taking advantage of the organotropism of bile acids. It has been demonstrated that conjugates of bile acids with amino acid L-alanine and a model tetrapeptide of alanine (ala_4_) were efficiently taken up by intestinal and hepatic cells, but in the case of 15 mer oligodeoxynucleotides an attached bile acid could not shuttle them successfully into the hepatocytes (Petzinger et al., 1999).

Nowadays, organotropism of bile acid-chemotherapeutic drug conjugates are being tested to overcome chemotherapy resistance of enterohepatic tumors and reduce side effects to healthy tissues by selectively targeting the drugs to tumor cells. First it was shown that four out of six synthesized chlorambucil-bile acid conjugates (S-2521, S-2539, S-2567, and S-2576) retained affinity toward ASBT transporter in the ileum, while conjugate S-2577 was able to alkylate proteins demonstrating the preserved drug character. Furthermore, liver perfusion experiments demonstrated that conjugate S-2576 is predominantly excreted by bile, unlike chlorambucil itself that is excreted by the kidney, which indicates that bile acids may deliver a coupled drug specifically to the liver or biliary tract if desired (Kramer et al., 1992). In nude mice, cisplatin-UDCA conjugate (Bamet-UD2) as a model ASBT-targeted drug, inhibited the growth of human colon adenocarcinoma tumors with induced stable expression of ASBT. Besides, as compared with cisplatin, administration of Bamet-UD2 to rats with cholangiocarcinoma resulted in an efficient liver and tumor uptake but low exposure of extrahepatic tissues to the drug (Lozano et al., 2015). Similarly, the potential application of bile acid-cytarabine conjugates for liver cancers has been investigated. Cytarabine has a poor oral absorption due to its rapid deamination and poor membrane permeability. Cytarabine conjugates with CA, CDCA, UDCA and hyodeoxycholic acid (HDCA) exerted potent antiproliferative activities against human hepatocellular carcinoma HepG2 cells. UDCA conjugate of cytarabine exhibited optimal stability *in vitro* and twofold increased oral bioavailability *in vivo* compared with the parent drug (Zhang et al., 2016).

## Toxicological Considerations of the Application of Bile Acids

The excipients of pharmaceutical dosage forms are exceptionally important part of a medicine since they exert key functions of guaranteeing the dosage, stability and bioavailability of the active principle. The components employed as excipients must present the characteristics required by their technological function, but also correspond to suitable safety requirements (Pifferi and Restani, 2003).

The safety concerns have been raised regarding the use of bile acids as pharmaceutical excipients, since accumulation and retention of hydrophobic bile acids have been implicated as a major cause of liver damage in cholestasis. Hydrophobicity is the most important determinant of cytotoxicity of bile acids, and hydrophobic bile acids such as DCA have been reported to exert tumor-promoting activity (Bernstein et al., 2011). Bile acids have the potential to induce cell death both through non-specific detergent (membranolytic) effects and through receptor-mediated interactions. They can promote the generation of reactive oxygen species that, in turn, oxidatively modify lipids, proteins, and nucleic acids, and eventually cause apoptosis or necrosis of cells. However, these effects are mostly produced when bile acids are present in supraphysiological concentrations (Perez and Britz, 2009). Besides, hydrophilic bile acids such as UDCA exert cytoprotective effects and can diminish toxic activity of hydrophobic bile acids (Amaral et al., 2009). Furthermore, it has been shown that phosphatidylcholine can prevent toxicity of bile salts on gastrointestinal epithelia and membrane, which may be related to the formation of less toxic mixed micelles (Barrios and Lichtenberger, 2000).

Several studies confirmed the ability of bile acids to induce mucosal damage and ciliotoxicity. As expected, DCA was proven to induce the most pronounced ciliary arrest compared to other bile salts (Lee and Yamamoto, 1989). Similarly, DCA at concentration of 1% caused ocular irritation and corneal damage, while other more hydrophilic bile salts did not induce these effects (Saettone et al., 1996).

The relative cytotoxicity of bile acids depends mainly on both structure of the bile acid and membrane properties, such as composition, lipid fluidity, charge and hydrophobicity (Garidel et al., 2007). Minor structural modifications of natural bile salts have led to the creation of bile acid derivatives with the reduced toxicity. Thus, it has been shown that replacing hydroxyl groups in CA with keto groups produces significantly less surface active and less lipophilic bile salts with diminished membrane toxicity. Much attention has been paid to the studies of MKC, a stable semisynthetic analog of CA, as absorption enhancer that has been proven to possess high permeation-enhancing activity and low toxicity (Mikov et al., 2018). On the contrary, the conjugation of bile acids with polyamines or cationic amino acids promotes electrostatic interactions with anionic cellular components, such as anionic membrane lipids or the phosphate backbone of DNA, and these cationic bile acid derivatives have been developed as potential anticancer, antimicrobial, and transfection agents in non-viral gene delivery (Singh et al., 2013).

## Conclusion

Research over the last several decades revealed the new functions of bile acids as absorption enhancers, which makes them suitable for designing novel drug delivery systems. The main advantage of bile acids is their ability to act as both drug solubilizing and permeation-modifying agents. Therefore, bile acids may improve bioavailability of drugs whose absorption-limiting factors include either poor aqueous solubility or low membrane permeability. Besides, bile acids may withstand the gastrointestinal impediments and aid in the carrier-mediated absorption of physically complexed or chemically conjugated drug molecules.

This review provides in-depth insight into the mechanisms of drug absorption enhancement mediated by bile acids, but also into the molecular interactions between drugs and bile acids that can impact absorption and pharmacokinetic properties of drug molecules. The studies presented here appear very attractive from a pharmaceutical point of view since these the most recent findings in the area of bile acid-based pharmaceutical formulations offer significant pharmacological options for the development of innovative drug delivery systems consisting of bile acids or their semisynthetic derivatives.

## Author Contributions

NP and MÐ reviewed the literature and drafted the manuscript. MM and HA-S conceived the idea. All authors NP, MÐ, BS, MM, SG-K, HA-S, and KS edited, revised, and approved the final version of this review.

## Conflict of Interest Statement

The authors declare that the research was conducted in the absence of any commercial or financial relationships that could be construed as a potential conflict of interest.
